# Supplementary material for: Biomechanical influences of gait patterns on knee joint: Kinematic & EMG analysis
Source: PLoS One. 2020 May 29;15(5):e0233593. doi: 10.1371/journal.pone.0233593 (PMC7259630; doi:10.1371/journal.pone.0233593)
Supplement: Attachment — Submitted filename: Response to reviewers.docx [file pone.0233593.s002.docx]

**Reviewer #1:**

Dear corresponding Author thanks for your submission. Please check these comments:
Q1. The age difference between the two groups is significant. How can you be sure that this difference is not the cause of different walking style? It is a sample problem.

- I agree with you. This study was performed prospectively. Participants above 50 years with or without spinal stenosis were consecutively included. Therefore, we could not match the age between the two groups.

In my opinion, old age, impaired proprioception, age-related weakness might also influence the results in the spinal stenosis group. But our study evaluated three different gait patterns such as normal, adducted and abducted gait pattern in each group. The differences in the three gait patterns in each group are thought to have contributed to the conclusion.

- I have added your comments in limitation section (page 18, line 337~339)

Q2. Please can you show information how you measured the stride width? You mean the horizontal distance between two feet? Or the stride length, in some part of the paper it is not clear.

- Thank you. For clarification, I have added the definition of stride width “Stride width was defined as the medial-lateral distance between the heels (Principles of Rehabilitation Medicine Ed, Raj, Mitra. New York, NY: McGraw-Hill)”. I also added figure 1(B, left image) on page 6, line 103~104.
- In abstract, “the higher stride” was replaced with “wider stride” for clarification on page 3, line 46, and page 3. I rechecked the whole manuscript for clarification of stride length.

Q3. The statistical approach is very simple and I don't think that it is correct to use the paired T-Test for the comparison of different way to walk in the same group. This kind of procedure is called "repeated measure" and so the statistical approach should be different. Usually some correction factors are used (such as Bonferroni). Please check this point with a statistic specialist just to be sure.

- Thank you. We consulted statistician and corrected as you recommended.
- We have corrected table 2. We divided right and left, bilateral and used repeated measure ANOVA and Bonferroni correction for comparison. In the control group, the left side was significantly changed at abducted gait (*). In the comparison of the two groups, the control group showed significant differences in normal gait pattern.
- In table 3, 4, we also used repeated-measures ANOVA and corrected the statistical analysis.
- We also have inserted “When measurements from normal gait were compared with those of abducted and adducted gait within the group, repeated measures ANOVA was used to compare the means. Bonferroni correction was used to reduce the risk of type I error.” In the Statistical Analysis section.
- To help understand the table, we reduced the special characteristics.

Q4. The results in table 2 are difficult to understand. Please can you explain how the system works to measure the FTA in varus and valgus. Moreover, the table 2 in not clear in terms of significance: there are two different symbols and it is not clear the comparison. Please modify.

- We used Human Track (Gait analysis training system) for femur tibia angle (FTA). This system uses five IMU sensors and stereo vision sensors to automatically calculate the three-dimensional joint angle and kinematic parameters. As shown in figure 1(A), five IMU sensors were attached at the abdomen (or lumbar), bilateral mid-thigh, mid-shank, and foot. As each IMU sensors have a gyroscope, magnetometer and accelerometer, the angle between two sensors can be measured.
- Thank you. I have added the explanations of the *, ^#.^

^#^: Comparisons of the normal gait pattern between the LSS and the control groups.

*: Comparisons of gait patterns within each group. *, ^#^: p-value < 0.05

Q5. The legend of figure 1A is wrong. The platform used is not a force plate but a pressure plate with a sensorized matrix.

- Thank you. I corrected as you suggested.

Q6. From the results it is not clear how did you use the stride width parameter. It is a very very interesting parameter but there is no explanation of the modification of this parameter in differents gait style. In the discussion paragraph you speak about stride width (line 259) but no data are presented in the results.

- For clarification, I added the definition of stride width at page 6, line 102 and Figure 1(B).
- The comparison of stride width is described in table 1 (normalized stride width). For comparison of normal, adducted and abducted gait, we defined three patterns as following (Figure 1(B, right image)). First, the participants were instructed to walk in their normal pace (“Normal gait”). Then, they were asked to walk with their medial borders of feet touching each other (“Adduction gait”). Finally, they were told to walk with their feet approximately 40cm apart (“Abduction gait”).

Table 2 describes the FTA and table 3,4 describes the sEMG values in three gait patterns.

- I have added “This software automatically demonstrated the stride width, step length, stride length and foot progression angle which is the angle between the heel and the path of gait.” In method section (Page 6, Line 104~106).

Q7. Line 281: which part of the results allows you to write this sentence? So the practical application of this paper, as you wrote in conclusion, is to suggest to walk with abducted legs because the higher muscular recruitment? Can you explain better this point? I understand that the muscle recruitment is higher for sure but the pain and discomfort as well. So it seems a strange suggestion.

- In the present study, the patients with spinal stenosis showed a wider stride width compared to the control group. Wider stride width, the activation of hip abductors compared to adductors increases, and femur tibia angle became closer to the varus. This distinctive gait pattern (abducted gait) exerts increased loading on the medial knee. Therefore, abducted gait pattern can be harmful to knee joints.

In my opinion, impaired balance and abductor weakness due to old age or spinal stenosis could contribute to abducted gait.

The present study suggests to strengthen abductor muscles and to improve balance to maintain normal gait pattern. But we didn’t measure the balance and abductor’ weakness, we didn’t mention the suggestions in the conclusion section.

**Reviewer #2:**

This paper requires a major revision. It is not coherent or methodologically sound (at least as described).

Q1. Putting aside the missing definite article in the title, the title itself is misleading, especially since they state in their discussion that they cannot make conclusions about knee OA.

- Thank you for the nice comment. I agree with you as our study couldn’t conclude the development of OA. Therefore, I removed “, relating to the escalated risk of knee osteoarthritis” and only mentioned increased loads on the medial knee on page 18, line 337~339.

Q2. The topic is poorly explained and background on both clinical and basic research is basically non-existent.

- Thank you for your comment. Though you mentioned poor background research,

These lacks of background knowledge imply our research is unique. As I mentioned in the manuscript, gait analysis was performed only in a sagittal plane. And no research using gait analysis combined EMG analysis was performed in the coronal plane. So this can be our research’s strength. As technology is improving, lots of researches can be done in the coronal plane after our research.

Q3. The hypotheses are vague, asserting weakness and its effect on gait and on the knee and on knee OA. They are not specific. Moreover, the argument for why this affects the knee rather than the hip is not stated clearly or convincingly. A causative effect is simply asserted and not explained in a way that makes it predictable.

- Thank you for your comments. Initially, I speculated LSS patients walk with narrow stride width because of the hip abductors’ weakness and less activation in gait. The wrong hypothesis implies there has been no background knowledge about gait analysis in the coronal plane. We wanted to find out the relationship of gait pattern, the degree of hip abductors’ activation and loading on the knee joint in the coronal plane. During analyzing our data, we realized the mechanisms of hip muscle and loading on the knee joint.
- To improve understanding and clarification, I have added figure 5 which explains these mechanisms.
- The Hip joint is relatively stable compared to knee points, and the incidence of arthritis is very low. So, the hip joint is thought to be less affected by the gait pattern than the knee joint. As our study didn’t include the kinematics of the hip joint, we didn’t mention that.

Q4. There is no real discussion in the introduction of the role played by abductors in normal gait and how the different gaits really worked. Is it not the case, that the patterns of EMG are very predictable across gaits. So this is really a question of comparisons between LSS and control. But even then the results are reasonably predictable.

- To improve understanding of the role of abductors in normal gait, I have added “In normal gait in coronal plane torque, adducting vector is maintained and the intensity of gluteus medius, minimus and tensor fascia lata muscles increases to a 20%, 20% and 25% of maximal manual muscle test value, respectively.(13)” in introduction section on page 5, line 77~79.(Reference: Gait analysis, Normal and pathological function. Jacquelin Perry)

Q5. I need clearer hypotheses and clearer statements about why this data is being pursued, aside from that it hasn't been collected before.

- The biggest reason for starting the study was that patients with spinal stenosis are commonly found to have knee arthritis. The second is that knee varus is very severe in patients with knee OA. Some older patients reach as high as 30 degrees. And a severe varus deformity was resulted from not only knee OA itself but also the biomechanical problem of the coronal plane. As our study didn’t include epidemiologic and longitudinal data, we only approached in the biomechanical aspect.
- To make clear the hypothesis, I have added the importance of hip abductor in gait cycle in the coronal plane and the reason why hip abductor is affected in severe LSS patients in introduction section (Page 5, line 77~79).

Q6. The adducted gait is intense and un-natural. I can only envision it as a shuffle. I could use more description of this gait to justify its inclusion.

- As I described in the method section, I defined adducted and abducted gait (page 7, Line 111~118). But shuffle is difficult to be used in a scientific paper. After checking the results of the study, we were able to draw conclusions from the study sufficiently as a result of the abducted and normal gait. However, when planning the study, both the adducted and abducted gaits were evaluated because the LSS patients who showed a hip abductors’ weakness expected to perform the adducted gait.

Q7. The choice of gaits leads to obvious associations. For example, it makes sense a priori that stride width would require more abductors. It also makes sense that stride width changes knee alignment.

- Thank you. The problem is that there has previously been a lack of research on the gait pattern and knee point loading. So I tried to verify these aspects.

Q8. How do you know you got EMG from lesser gluteals. I believe you, but would like to know how you verified that was the case in detail.

- In surface EMG study, cross-talk occurs at all times. Though gluteus medius was targeted, glutes maximus and minimus muscles worked together. And their activations also affect EMG sensors. However, in this study, three gat patterns were studied in the same position without removing or reattaching the electrodes, therefore, bias could be minimized. Also, we used a reference position to minimize the crosstalk phenomenon (Cram JR. Cram's introduction to surface electromyography: Jones & Bartlett Learning; 2010.)

Q9. I have no idea why the EMG was normalized to QF. Please explain the logic for this. It doesn't make sense to me at this point. I also worry that some of the patient group has OA already and that QF weakness and OA are correlated. So this may lead to weir values.

- Normalization is always an issue in SEMG research. In the initial planning period, we thought that there would be a difference between the RMS values and the peak amplitude values in the LSS patients and normal participants. So, we need to compare the differences. Initially, we thought the amplitude of the quadriceps muscle would be stable during the normal, adducted and added gait patterns, so we planned to use the ratio of hip abductor to the quadriceps value. Interestingly, our results showed that the QF muscle’s activation was also increased like hip abductors in the LSS group and decreased in the control group (Table 3). But the increase was smaller than the hip abductors. Therefore, we decided to use the proportion of the value as it is to emphasize the relative activation of hip abductors in abducted gait pattern (Table 4).

Q10. Much of the discussion addresses complexities and contradictions in the results. But these challenges are unexpected to the reader because they are poorly laid out in the introduction.

- To make an understanding of the role of abductors in normal gait, I have added an explanation of normal gait in the aspect of torque and vector in the instruction section on page 5, line 77~79. Due to a limited number of words, a more detailed explanation is difficult. I hope you understand.

Q11. The discussion does a more thorough job setting up the problem and much of it could have appeared in the intro. But also in the discussion we learn that the data necessary to test at least one of the hypotheses has not been collected. "Therefore, the causality between knee osteoarthritis and LSS cannot be drawn from this study." This was a purpose of the study.

- I agree with you that the development of OA cannot be drawn from the results. Only increased loading can be drawn from the study. Therefore, I modified the conclusion.

Q12. Figure 4 seems to be mislabeled.

- Thank you very much. I corrected the mistake. I also corrected the legend of figure 4.
