# Supplementary material for: Biomechanical influences of gait patterns on knee joint: Kinematic & EMG analysis
Source: PLoS One. 2020 May 29;15(5):e0233593. doi: 10.1371/journal.pone.0233593 (PMC7259630; doi:10.1371/journal.pone.0233593)
Supplement: Attachment — Submitted filename: Response to reviewers_v2.docx [file pone.0233593.s003.docx]

1) the age problem in the sample is still present

- I agree with your comment. The difference in mean age between the LSS group and the control group could be a limitation of our study. We have additionally made a comment on this matter in the “Study limitation” section.
- We have added “A follow-up study is required to evaluate the stride width according to age.”

2) I know the sotfware you use and it doesn't measure the width, moreover it is not showed in the figure 1A as you write.

- The raw data of the contact points made during each gait cycle was exported as Microsoft Excel file(.XLS format) according to manual specified by the FreeStep program. The width of each cell was 0.5cm. By counting the medial-lateral distance between the heels in terms of cell number, we could obtain the stride width. We have modified the Figure 1 and Figure 1 legend. Thank you.
